# Cost-effectiveness of the rural lifestyle eating and activity program (Rural LEAP)

**DOI:** 10.1371/journal.pone.0326383

**Published:** 2025-07-16

**Authors:** Tiffany A. Radcliff, Murray J. Côté, Meena N. Shankar, Patricia E. Durning, Kathryn M. Ross, David M. Janicke, Christie A. Befort, Laurel S. Curran, Michael G. Perri

**Affiliations:** 1 School of Public Health, Texas A&M University, College Station, Texas, United States of America; 2 College of Public Health and Health Professions, University of Florida, Gainesville, Florida, United States of America; 3 University of Kansas Medical Center, Kansas City, Kansas, United States of America; Wingate University, UNITED STATES OF AMERICA

## Abstract

Rural United States (U.S.) residents with obesity have unique challenges maintaining successful weight loss; tailored support resources such as individual behavioral coaching through telehealth are a cost-effective option. This study examined the cost-effectiveness of telephone-based individual coaching sessions for extended care after initial weight loss compared to an education-only control group. Trial data collected during a randomized trial conducted in rural counties in Florida from October 21, 2013, to December 21, 2018 informed the base case parameters. Using a program/participant perspective, cost-effectiveness was assessed for a 5-year time horizon using a discrete Markov model, with sensitivity analysis to test model assumptions. A 3% discount rate was used to value future time periods, and prices were inflated to 2024 values. Primary endpoints were the proportion of participants in each weight loss category, measured as a < 5%, 5–10%, or >10% reduction from baseline weight. Cost-effectiveness was assessed using program costs and participant-reported Quality-Adjusted Life Years (QALYs) and health care costs. Incremental cost effectiveness ratios (ICERs) were calculated for the end of the trial and 5 years post-trial. Cost-effectiveness used a willingness-to-pay threshold of $150,000/QALY. Results identified that intervention with 18 individual telephone counseling sessions was more expensive than the education/control program to deliver ($555/participant vs. $27/participant) but also more effective (34.2% with at least 10% weight loss vs. 17.0% for control at the end of the intervention), with 6.4% of modeled participants expected to maintain at least 10% in baseline reduction at year 5, compared to 5.4% for controls. Intervention participants were predicted to have modestly lower out-of-pocket prescription and other medical costs compared to participants in the control group, which offset some of the incrementally higher coaching program costs. Predicted ICERs at 5 years ranged from $7,731 to $8,156 per QALY gained through the individual coaching program. Findings contribute to evidence needed to identify cost-effective strategies for long-term weight management and disease prevention for at-risk populations.

## Introduction

The prevalence of obesity in the United States (U.S.) has increased markedly since 2000, rising from approximately 30% to more than 40%. The current prevalence of obesity in the U.S. stands at 40.3% [1]. Moreover, the prevalence of obesity is higher in rural (nonmetropolitan) areas than in urban (metropolitan) areas. Data collected from 2013–2016 showed a prevalence of 43.1% among adults living in nonmetropolitan statistical areas compared with 35.1% for those living in large metropolitan statistical areas [2]. Obesity remains a high priority health risk in the U.S., with steadily increasing rates of adults observed with overweight or obesity over the past six decades and higher rates of obesity reported for rural residents [3–16]. Rural residents of the United States have been identified as a population at greater risk of obesity compared to urban residents, with barriers in the rural infrastructure and socioeconomic factors that can reduce access to affordable healthy foods, physical activity, and healthcare services [17–19]. Obesity increases the risk of developing heart disease, stroke, type 2 diabetes, fatty liver disease, osteoporosis, and certain cancers [20]. From both a clinical and public health perspective, these trends are concerning due to known higher risks of illness and injury due to weight-related health conditions and lower rates of health insurance for rural residents [3–6,10,21]. Fortunately, evidence-based interventions that result in effective weight loss are increasingly available, with options such as semaglutide 2.4 mg injections that cost patients without insurance around $1,350 per month, bariatric surgery for $17,000-$26,000, or memberships in behavioral change programs that emphasize lifestyle changes to improve diet and physical activity such as WeightWatchers®, Noom, and Wondr that promote healthier diets and more physical activity for members with fees that average $20-$70 per month [22]. Even with a growing array of options and resources to support healthy weight loss, there remains an ongoing challenge to identify cost-effective ways for individuals to maintain healthier weights and associated health benefits once the initial weight loss is achieved.

Prior research has identified that successful approaches to address challenges of obesity in rural areas requires multi-faceted strategies that combine healthy diets, fitness, education, and reducing barriers to healthy choices required for long-term lifestyle maintenance [7,8,18,23–26]. A series of trials have leveraged the expertise of the United States Department of Agriculture’s Cooperative Extension Service (CES) to support rural residents with obesity who are motivated to lose weight and test various modalities for sustaining lifestyle change [18,25–27]. For example, the Rural Lifestyle Eating and Activity Program (Rural LEAP) trial identified that individual coaching sessions delivered by telephone were comparatively more effective than the control condition in supporting sustained weight loss, but group coaching sessions using the same telephone modality were not [27]. As such, this analysis used the Rural LEAP trial data to inform the cost-effectiveness of individual telephone behavioral coaching compared to the education control group.

## Methods

### Analytical overview

The Rural Lifestyle Eating and Activity Program (Rural LEAP) trial (ClinicalTrials.gov identifier: NCT02054624) was designed to study options that may help rural adults with obesity manage their weight and increase physical activity, with an important goal of identifying which type of follow-up program was most effective in reducing weight regain after successful weight loss. The study was approved by the Institutional Review Board at the University of Florida.

The economic analysis followed the plan proposed in the funding application and used current Consolidated Health Economics Reporting System (CHEERS 2022) to guide reporting. A trial-based analysis and a Markov model using discrete time steps and transition probabilities that varied by treatment group and over time was developed in Microsoft Excel® (MS Windows 365) to assess the cost effectiveness of individual coaching via telephone compared to an education control group in maintaining a clinically-meaningful weight reduction at the end of the trial [28,29]. Secondary outcomes included participant-reported health status and participant-reported monthly medication and other healthcare costs that were collected through participant surveys that included the Short Form 36 item (SF-36) general health questionnaire from the Medical Outcomes Study [30], a visual analog measure of health status using a health thermometer image for self-rating [31], and trial-specific questions on patient healthcare costs at baseline and the end of each phase of the trial. Trial participants were modelled for an additional five (5) years following the trial, which represents a timeline that is associated with successful long-term weight loss and aligns with other studies that model weight loss [32]. During the modeled follow-up timeframe, participants could maintain or continue their initial weight loss, regain some or all the initial weight lost, or die from all causes. Mortality rates were estimated using age and sex-adjusted life tables from a published analysis of the impact of obesity on all-cause mortality [33]. The Markov model cycle length, or time between state transitions, was 6 months. Model inputs and characteristics of the study population from the trial are listed in Table 1.

**Table 1:** Trial participant characteristics and model assumptions.

Participant Characteristics	Base Case Parameters	Std. Deviation	Source/Notes
Sex (% of Participants)			Rural LEAP Trial data
Female	82.7%		
Male	17.3%		
Age (Average, years)	55.4	10.3	Rural LEAP Trial data
Weight (kg) at month 0 (phase 1)	99.8	14.6	Rural LEAP Trial data
**Other Assumptions and Parameters**			
*Weight Loss Outcome Categories*	% Reduction		Participant Weight (kg) was measured at the beginning of the trial (month 0) and at the end of each trial phase
Limited/No Weight Loss vs. month 0	< 5%		
Moderate Weight Loss vs. month 0	5-10%		
Large Weight Loss vs. month 0	> 10%		
*Mean participant-reported health status by % Weight Loss Category*	Visual Analog Scale Score	SF-36 General Health Score	Rural LEAP Trial data
< 5%	64.71	61.94	Survey responses observed in Trial data at month 16 (end of phase 2)
5-10%	73.73	73.86	
> 10%	82.96	79.68	
*Mean participant-paid healthcare expenses by Weight Loss Category*	Monthly Medication $	Monthly Other Healthcare $	Rural LEAP Trial data, Adjusted to 2024$
< 5%	$44.97	$57.37	Self-reported responses observed in Trial data at month 16 (end of phase 2)
5-10%	$40.71	$33.69	
> 10%	$35.20	$43.81	

Note: Parameters were calculated using data from all Rural LEAP trial participants (n = 445) eligible for phase 2, including 143 participants assigned to the “Group” coaching intervention, which was previously identified as no more effective than the education control group, and therefore excluded from the cost-effectiveness analysis. Missing responses in survey data received from participants were assumed to be missing at random.

### Study population

Participants in the Rural LEAP trial underwent screening for eligibility at local Cooperative Extension Service (CES) offices in select rural counties in Florida. Cooperative Extension Service (CES) offices are located in each County and are based on partnerships with land-grant universities to provide research-based information to improve agriculture, nutrition, and the environment in local communities. The trial included 528 adult participants for a single evidence-based program for Phase 1 (active weight loss), with 445 active participants from Phase 1 eligible for randomization to a weight maintenance arm in Phase 2. Phase 1 eligibility required participants to be ages 21–75 with a body mass index (BMI) between 30 and 45 kg/m2 without medical contraindications for weight loss. As previously reported, no statistically significant differences in demographic characteristics (i.e., gender, ethnicity, education, or income) or initial (Phase 1) weight change were observed between groups assigned in Phase 2 [27]. Trial participants who completed at least half of the Phase 1 sessions were randomly assigned to a Phase 2 group. Phase 2 participants assigned to individual telephone coaching sessions received biweekly contact from a trained interventionist for 6 months and then monthly contact for 6 months for 18 coaching sessions where they received tailored guidance to problem-solve and overcome obstacles to maintaining their initial weight loss and were instructed to continue self-monitoring their food intake and step counts to sustain their goals. At each scheduled contact point during the 12 months of Phase 2, all participants, including those assigned to the education control group, received a weight management module delivered via e-mail (or via US mail by request) that included information on behavioral activities targeting weight maintenance. Phase 3 was a 6-month window during which participants were encouraged to continue managing their weight, but without any contact from the study, with a final assessment at the end of that timeline [27].

### Economic analysis details

The analysis used the perspective of the program/participant to identify relevant costs. Weight and other participant outcomes were measured during the Rural LEAP trial at baseline (month 0), month 4 (end of Phase 1), month 16 (end of Phase 2), and month 22 (end of Phase 3). Weight in kilograms was measured using a calibrated clinical scale at each timepoint, with multiple imputation methods used to replace missing weight values [27]. Participant health status was assumed to depend somewhat on successful weight reduction and used participant-reported survey responses to a visual analog scale (HT-VAS) rating (0–100 scale) and Short-Form 36 (SF-36) general health questions (0–100 scale) along with participant weights collected during the trial to confirm this relationship (see Table 1) [34]. Participant surveys at the end of trial phase 2 identified self-reported monthly medication and other healthcare costs, which were used to calculate expected monthly costs for each weight loss category (see Table 1).

Program delivery and participant costs were collected by the study team during the trial. Incremental costs during phase 2 were examined in relation to intervention outcomes since all participants received the same active weight loss program during Phase 1 and there were no intervention activities for participants during Phase 3. Main categories of costs for the individual coaching intervention included program staff time and wages to prepare for and deliver coaching sessions, materials provided to participants and coaches, and conference call services, all of which were tracked by the research team during the trial. Participants randomized to receive the coaching intervention called into 18 scheduled sessions with their health coach that lasted 10–20 minutes. Call-in sessions included a review of progress toward goals, weight management modules, tailored problem solving, and an action plan to set goals for the next session. Health coaches logged their time for calls, including preparation and follow-up activities. Phase 2 costs for the control group were limited to program staff preparing and sending 18 weight management modules delivered via e-mail (or via US mail by request). Program costs per participant were calculated by dividing the total costs by the number of participants.

All costs were adjusted to constant 2024 dollars using the U.S. Bureau of Labor Statistics’ Consumer Price Index (CPI) inflation calculator. A discount rate of 3% was used to assess future costs and outcomes relative to present values.

### Markov model and simulation overview

**Fig 1:**
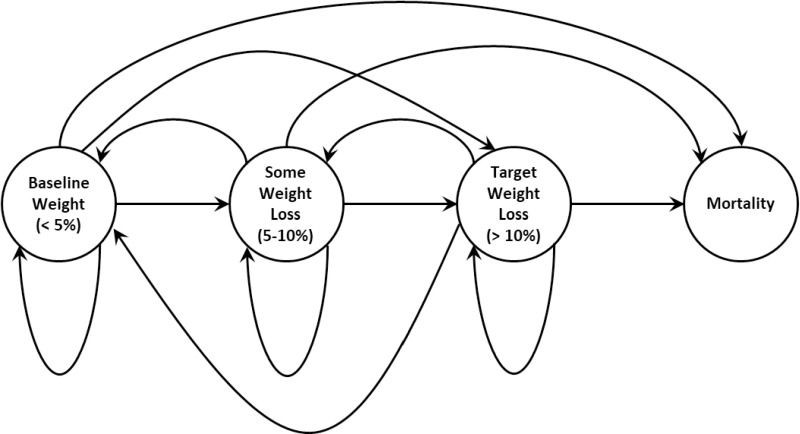
Conceptual Markov model. This figure identifies transitions to and from three weight loss categories, less than 5% weight loss, 5-10% weight loss, and greater than 10% weight loss that are possible in the Markov model. Mortality based on age and gender characteristics of the trial participants is included as an absorbing state. For a given time period, participants can remain in the same category or transition to any of the other categories, which depends on the estimated transition probabilities from the Rural LEAP trial.

A discrete time Markov model was constructed to represent participant transitions between and among the weight loss categories. Fig 1 illustrates the Markov model transitions across weight loss categories that were selected to capture clinically significant weight loss results, with less than 5% weight loss denoting limited or no weight loss compared to baseline (month 0), 5–10% weight loss representing moderate weight loss compared to month 0, and >10% for large weight loss compared to month 0. The thresholds were selected to align with published percent weight loss recommendations aligned with distinct health benefits [35]. Nodes (circles) represent each participant’s state while the arcs (arrows) indicate the direction of possible transitions. For example, participants with “Limited or No Weight loss” remain in that state if they did not have a weight in kilograms that was at least 5% lower than the beginning of phase 1, transition to the “Moderate weight loss” state (5–10% reduction compared to their baseline weight), transition to the “Large weight loss” state with greater than 10% reduction compared to their baseline weight, or transition to mortality (i.e., die) after a year. Other nodes and arcs can be interpreted similarly. Each participant’s probability of transition across states depended on their current state and age-related probability of mortality. The probabilities were estimated as the proportion of participants who transitioned into a future state over the number of participants in a current state. Participants move across all other categories at six-month transitions with mortality as the only absorbing state, meaning that once a participant is in this state, they remain in that state. Within-trial observed outcomes were used to inform the transition probabilities and expected number of participants in each weight loss category for trial months 0–22. Beyond that timeframe, the model uses the last observed transition probabilities (i.e., the transitions that were observed in trial month 22) to predict transitions through a five-year horizon and maintain a conservative estimate of participant transitions between and among the weight loss categories. The estimated six-month transition probabilities were converted to estimated annual transition probabilities using standard approaches [36]. Mortality/loss to follow up was assumed to be the same for all participants, regardless of their assignment to individual coaching or education control. Averages of self-reported health ratings from the SF-36 responses and visual analog scale, with scores that could range from 0 (lowest health) – 100 (best health), were used as alternative measures for Quality Adjusted Life Years (QALYs). This information was compared with averages of self-reported healthcare costs across each weight loss category at the end of Phase 2 to identify outcomes of the individual counseling and education control modelled for a 5-year follow up timeframe (see Table 2) using a 3% discount rate.

**Table 2:** Total and per-participant program delivery costs by trial phase, in constant January 2024 U.S. dollars.

	Education Control	Individual Coaching
	n = 153	n = 149
Trial Phase 1 (Months 1–4) – Active Weight Loss Phase		
Program Staffing	$38,225.79	$37,226.42
Program Manuals	$5,246.83	$5,109.66
Intervention Materials	$6,271.12	$6,107.17
Total Phase 1 Delivery Costs	$49,743.74	$48,443.25
**Total Phase 1 Delivery Cost Per Participant**	**$325.12**	**$325.12**
Trial Phase 2 (Months 5–16) – Weight Maintenance Intervention Phase		
12-month Program Staffing	$4,085.13	$80,830.13
12-Month Teleconference Service	$-	$1,911.06
Total Phase 2 Delivery Costs	$4,085.13	$82,741.19
**Total Phase 2 Delivery Cost per Participant**	**$26.70**	**$555.31**
Total Program Costs for Trial Phases 1 and 2		
Total Phase 1 and Phase 2 Delivery Costs	$53,828.87	$131,184.44
**Total Phase 1 and Phase 2 Delivery Cost per Participant**	**$351.82**	**$880.43**
Incremental Difference (Individual Coaching Intervention vs. Education Control)		**$528.61**

## Results

### Observed trial costs and outcomes

#### Program delivery costs.

All participants were enrolled in the same eating and activity program during phase 1, resulting in the same average cost per participant in phase 1 regardless of phase 2 group assignment. Participants assigned to the individual coaching intervention had observed inflation-adjusted per-participant costs of $555.31 for the 12-month coaching intervention in phase 2, compared to $26.70 for participants in the control group (see Table 2). Due to the nature of the coaching intervention, most (over 75%) of the individual coaching intervention costs were for program staffing to deliver the sessions and materials to participants, with the remainder used for teleconference call services. With 18 coaching sessions per participant in the intervention, the average cost per session was around $31 ($555.31 ÷ 18). Phase 2 delivery costs for the control group were primarily for program staffing to distribute program materials.

**Fig 2:**
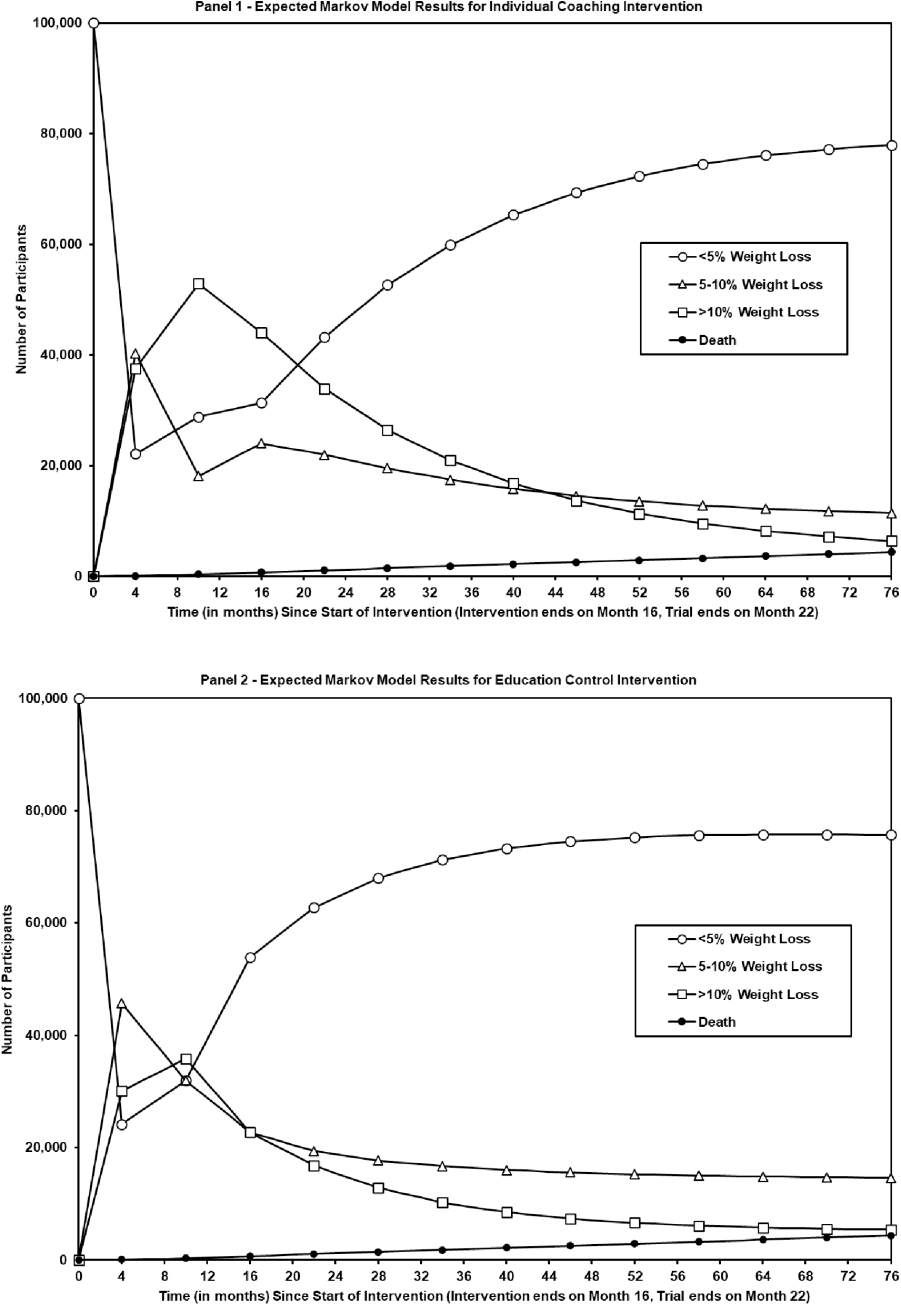
Expected Markov model results by intervention. The two panels illustrate the expected flow of 100,000 participants in the individual coaching intervention (panel 1) from month zero (trial enrollment) or education control group (panel 2) through trial phase 1 (end of month 4), trial phase 2 (end of month 16), and trial phase 3 (end of month 22), with expected follow-up to month 70. The model uses transition probabilities observed during the trial to generate expected percentages of participants in each weight loss category over time.

Participants assigned to the individual coaching intervention had higher observed percentages of both moderate (5–10%) and high (>10%) weight loss at the end of trial phases 2 and 3 compared to participants in the education control (see Table 3 and Fig 2), and average self-reported monthly out-of-pocket medication and healthcare costs for participants with less than 5% weight loss were higher ($102.33/mo) compared to those with 5–10% ($74.40/mo) and >10% weight loss ($79.01/mo).

**Table 3:** Weight outcomes observed in the trial and modeled for 5 years.

	<5% Weight Loss	5-10% Weight Loss	>10% Weight Loss	Mortality/Loss to Follow up
**Month 16 (End of Phase 2)**				
Individual Coaching	31.5%	24.2%	44.3%	0.0%
Education Control	54.3%	22.9%	22.8%	0.0%
**Month 22 (End of Phase 3)**				
Individual Coaching	43.6%	22.2%	34.2%	0.0%
Education Control	63.4%	19.6%	17.0%	0.0%
**Modeled Results – 5-year Horizon**				
Individual Coaching	77.9%	11.4%	6.4%	4.3%
Education Control	75.7%	14.6%	5.4%	4.3%

#### Modeled costs and outcomes.

Fig 2 (panels 1 and 2) show the expected results for a hypothetical 100,000 participant cohort under the control group and individual telephone counseling. The distribution of participants by state (i.e., < 5% weight reduction, 5–10% weight reduction, > 10% weight reduction, and Mortality) are governed by the state-dependent transition probabilities calculated within the Markov model. The estimated transition probabilities are time dependent as they are not assumed to remain constant across the observed time increments in the trial data. For the 5-year time horizon, the estimated annual transition probabilities were based upon the estimated transition probabilities determined at the end of trial phase 2 (month 16) when active engagement ended.

The Markov model predictions are consistent with the within-trial behavior previously published [27]. Specifically, month 16, it was observed that not all participants were able to maintain their weight loss and regained some or all of the weight lost, which results in a the “dip” in the expected number of participants in the > 10% Weight Loss category between months 16 and month 22. After month 22, the Markov model estimates the expected number of the 100,000 hypothetical participants that would be in each category for up to five years after the intervention.

The Markov model with a 5-year time horizon predicted that approximately 6.4% of the individual telephone coaching participants would maintain >10% weight reduction at 5 years compared to 5.4% for the education control group (see Table 3). However, education control participants had a higher probability of moderate (5–10%) weight loss predicted at 5 years, 14.6% compared to 11.4% in the intervention group (see Table 3). This predicted result is largely due to the observed behavior of this group at the end of phase 3 (month 22), when control group participants had a slightly greater likelihood of transitioning into moderate weight loss compared to participants who received individual Ob t (the observed transition probability from >10% weight reduction to moderate weight loss was 0.2571 for the control group and 0.2273 for individual coaching). Participants in the individual coaching intervention were predicted to have higher self-reported QALYs using either the SF-36 or HT-VAS measures compared to the education control group at both the end of the trial and over 5 years. The incremental gain was around 0.04 QALYs over 1 year (14–15 more healthy days) to around 0.06 QALYs over 5 years (20–21 healthy days, see Table 4). Total costs, including both the program delivery costs and out of pocket healthcare spending, were modestly higher for the coaching intervention participants at the end of the trial (month 22), with an incremental difference of about $464 over 12 months, and $446.50 over the modeled 5-year time horizon (see Table 4). These reduced incremental costs over time reflect the lower self-reported monthly out-of-pocket healthcare costs for participants with more than 10% weight loss, which was more likely for individuals assigned to the coaching program.

**Table 4:** Cost-effectiveness of individual coaching: End of trial and 5-year simulation model.

Phase 2 Assignment	Participant Outcomes End of Trial (Month 22)			Expected Participant Outcomes (Post Trial, 5-year Time Horizon)		
	Costs Per Participant	QALYs (HT-VAS)	QALYs (SF-36 GH)	Costs Per Participant	QALYs (HT-VAS)	QALYs (SF-36 GH)
Individual Coaching	$1,578.37	0.7497	0.7268	$5,774.80	3.1761	3.0652
Education Control	$1,114.01	0.7095	0.6872	$5,328.30	3.1183	3.015
**Incremental Difference** (Individual – Education)	$464.36	0.0403	0.0395	$446.50	0.0578	0.0547
**Incremental Cost Effectiveness Ratio (ICER)**		**$11,533.36**	**$11,746.44**		**$7,730.51**	**$8,155.83**

### Cost effectiveness

Table 4 presents the ICER estimates that were observed at the end of trial Phase 3 and modeled for a 5-year horizon after the trial. Costs per participant included program costs (Table 3) plus monthly average medication and other healthcare costs that were self-reported. Participant healthcare costs for each individual were assigned according to weight loss category (<5%, 5–10%, and >10% weight loss) reported in Table 1. QALYs were calculated using the HT-VAS and SF-36 GH at the end of the trial (observed at month 22) and projected over 5 years, with possible score ranges of 0–1.0 QALYs or 0–5.0 QALYs, respectively. Participants in the individual coaching program had both higher costs and higher QALYs, which indicated the need for an ICER analysis. The ICER calculations ranged from $11,533 to $11,746 per QALY at the end of trial (month 22) and from $7,731 to $8,155 for the 5-year horizon, depending on which QALY measure is used (see Table 4). Further, because respondents with >10% weight reduction reported lower medication and other out of pocket healthcare costs, coaching intervention participants were expected to have modestly lower out of pocket medication and other healthcare costs compared to the control group ($5,115 vs. $5,437), which offset some of the higher coaching program costs over the modeled 5 year time horizon.

### Sensitivity analysis

**Fig 3:**
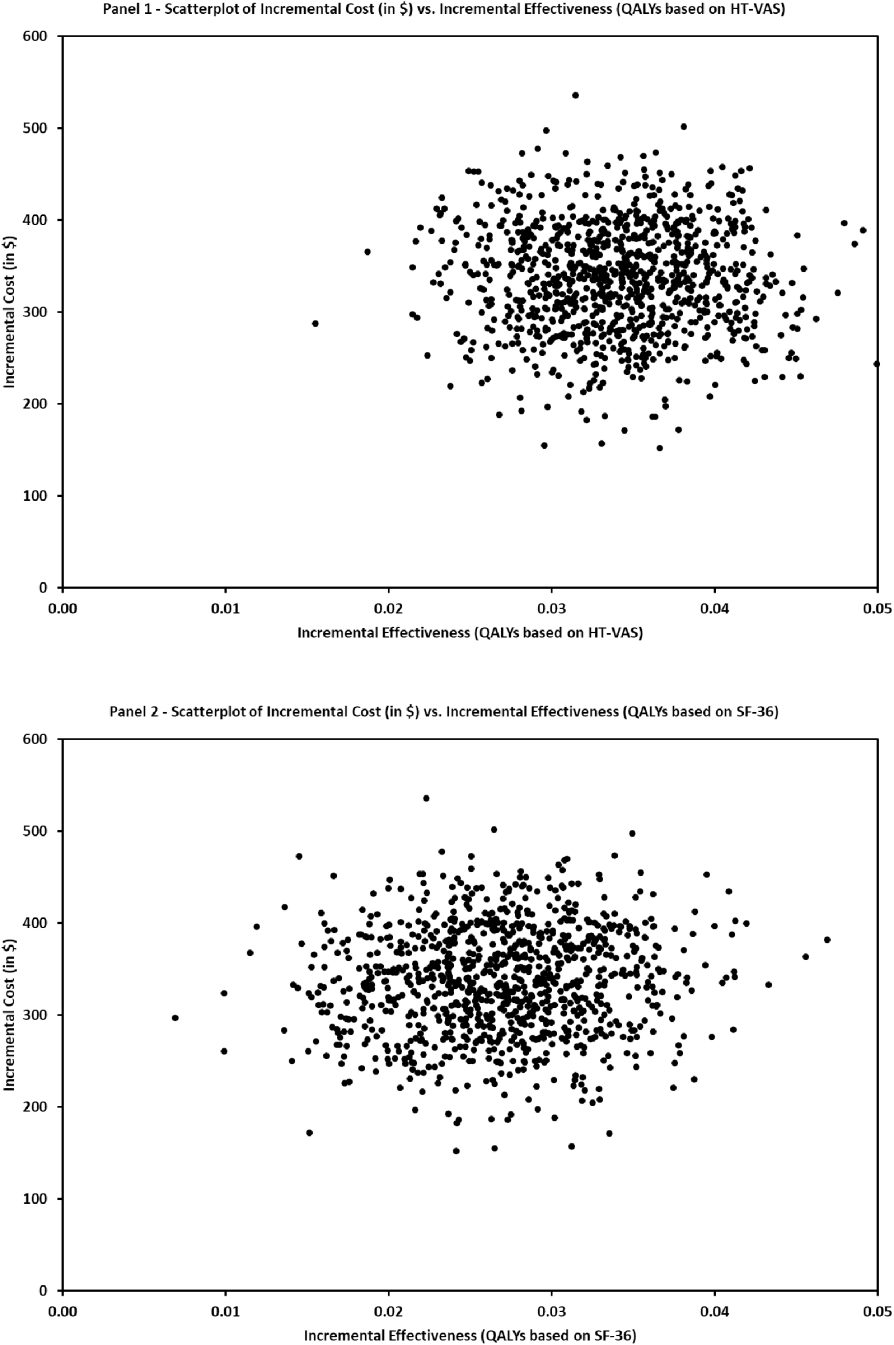
Probabilistic sensitivity analysis by QALY measurement. The two panels illustrate the results from 1,000 simulated replications of the Markov model for the incremental effectiveness measured by QALYs using HT-VAS (panel 1) or SF-36 (panel 2) and incremental cost in $.

To determine the robustness of the Markov model and its expected value, a probabilistic sensitivity analysis (PSA) and one-way sensitivity analysis were performed. MatLab’s “fitdist” function was used to identify the best fitting probability distributions of key parameters from the base-case [37]. Results identified that a truncated normal distribution could reasonably model the self-reported HT-VAS and SF-36 measures since these measures are bounded between 0 and 100. The self-reported costs for monthly medication and other healthcare expenses were modeled with a mixed distribution that captured both $0 expenses and>$0 expenses. The relative frequency of the observed $0 expenses was used as the probability of that occurrence while a gamma distribution was used for>$0 expenses. The Markov model was then simulated for 1,000 trials to determine how the ICERs would vary. Fig 3 displays two scatterplots that illustrate the possible variability between incremental effectiveness measured by QALYs using HT-VAS (panel 1) or SF-36 (panel 2) and incremental cost in $. In over 99% of the 1,000 replications, the individual coaching was cost effective with similar results for the two measures used for QALYs.

Beyond the PSA, we examined the sensitivity of key parameters in the Markov Model using a one-way sensitivity analysis as shown in Table 5. We tested sensitivity of the results to assumptions regarding program costs, self-reported monthly medication, other healthcare expenses, and total expenses. Following other studies, we varied these parameters by ±20% while the discount rate was varied from 3% to 1% or 5% [38–40]. Both the PSA and the one-way sensitivity analysis did not change the main outcomes.

**Table 5:** One-way sensitivity analysis on expected participant outcomes (Post trial, 5-year time horizon).

					Incremental QALY		ICER	
	Component	% Change	New Cost	Incremental cost	HT-VAS	SF-36 GH	HT-VAS	SF-36 GH
Base case (from Table 4)				$335.08	0.0342	0.0270	$9,797.86	$12,416.15
Phase 2 Program Costs by Treatment Arm	Individual	20%	$666.37	$440.81	0.0342	0.0270	$12,889.07	$16,333.55
	Education	20%	$32.04					
	Individual	−20%	$444.25	$229.36	0.0342	0.0270	$6,706.50	$8,498.74
	Education	−20%	$21.36					
Average Monthly Prescription Cost by Weight Loss	< 5%	20%	$53.22	$258.33	0.0342	0.0270	$7,553.53	$9,572.13
	5-10%	20%	$50.89					
	> 10%	20%	$42.07					
	< 5%	−20%	$35.48	$411.84	0.0342	0.0270	$12,042.04	$15,260.16
	5-10%	−20%	$33.93					
	> 10%	−20%	$28.05					
Average Monthly Healthcare Cost by Weight Loss	< 5%	20%	$70.22	$327.49	0.0342	0.0270	$9,575.81	$12,134.86
	5-10%	20%	$41.45					
	> 10%	20%	$51.91					
	< 5%	−20%	$46.82	$342.68	0.0342	0.0270	$10,019.75	$12,697.44
	5-10%	−20%	$27.63					
	> 10%	−20%	$34.61					
Average Total Monthly Cost by Weight Loss	< 5%	20%	$123.44	$250.74	0.0342	0.0270	$7,331.55	$9,290.84
	5-10%	20%	$92.34					
	> 10%	20%	$93.98					
	< 5%	−20%	$82.30	$419.43	0.0342	0.0270	$12,264.01	$15,541.45
	5-10%	−20%	$61.56					
	> 10%	−20%	$62.66					
Discount Rate		1%		$328.44	0.0351	0.0277	$9,351.99	$11,867.45
		5%		$341.20	0.0333	0.0263	$10,231.79	$12,949.27

## Discussion

Resource availability, program costs, participant healthcare costs, and cost-effectiveness are important considerations for assessing the value of weight management programs and related adoption by rural communities seeking feasible options to support their residents. This analysis used trial-based data to assess the costs and cost effectiveness of individual coaching by telephone compared to an education control. A Markov model using trial-based inputs extended the analysis to estimate 5-year outcomes, costs, and cost-effectiveness. Both with within-trial estimates and 5-year model identified that 18 individual telephone counseling sessions were cost-effective. Individual coaching sessions costs were around $31 per session, which is aligned closely with prices of commercially available weight loss programs and likely affordable for many rural adults who are motivated to initiate or maintain weight loss with behavioral support programming. Further, the cost per QALY gained through the intervention were modest, reflecting the low incremental costs of the coaching program along with the higher probability of coached participants to maintain at least 10% weight reduction and self-report gains in health status. Both the end of trial analysis and simulation identified modest improvements in weight and health status that result in a favorable ICER compared to the education control. Further, a sensitivity analysis determined that a 20% increase in the coaching intervention costs would still result in ICERs below $150,000/QALY threshold selected to identify cost-effectiveness.

Results align closely with existing literature but offer additional insights on populations that are both rural and in the southeastern U.S., where obesity rates are consistently higher than in other parts of the nation. Findings imply that clinically significant weight losses achieved by adults in rural communities can be maintained via a cost-effective program of individual telephone counseling. This study is one of the first randomized controlled trials to address the issue of how to sustain weight losses achieved by adults who reside in rural communities. The results highlight how follow-up care provided via a cost-effective program of individual telephone counseling can benefit participants. The study participants were drawn from rural communities in the southeastern U.S., thereby limiting generalizability to adults in other geographic areas. The modest projected cost savings of telephone counseling compared to other modalities for behavioral coaching represents an additional limitation. Results represented in the cost-effectiveness analysis reflect the characteristics of adults eligible for the Rural LEAP trial and may not be representative of other rural adults or other adults with obesity. Our study population was 81% female, a percentage not dissimilar to the mean of 73% female observed in a comprehensive review of weight-loss trials [41]. Women are more likely than men to volunteer for weight-loss studies, and this trend may be heightened in rural communities where social norms make it less likely for men to seek treatment for obesity [42]. However, the behavior modification programs used by the trial were based on well-supported evidence of efficacy [43–45]. Costs of the individual telephone counseling program identified in the trial were modest but included the critical teleconferencing services and personnel costs of leading the sessions; prior research has confirmed that telephone-based interventions are less expensive than face-to-face treatment and are equally effective [25,46]. Results presented in Table 3 demonstrate key assumptions used in the Markov model projections; namely that the last observed transition probability was used for all subsequent predictions and may not accurately reflect actual weight loss categories that would be observed in 5 years after the intervention. Future studies or programs may wish to examine feasibility of continued coaching at intervals beyond those examined in the Rural LEAP trial to determine if coaching sessions can promulgate sustained results for participants who initially lose weight.

The recent success of glucagon-like peptide-1 (GLP-1) medications in supporting rapid weight loss has garnered substantial popular attention, particularly as new studies identify reductions in cardiovascular risk and other major health issues associated with obesity [47]. Alongside the evidence of clinical benefits, however, have been concerns that stopping these medications is associated with weight regain for patients who have not adopted lifestyle changes that promote long-term health [48]. Further, access to medical and surgical interventions is beyond the reach of many rural Americans who may not have insurance or providers that can support these treatments [49]. Thus, the importance of cost-effective behavioral change programs tailored for specific populations remains highly relevant for clinicians and payers. Results in this study indicate that individual telephone counseling represents a cost-effective option for sustaining initial weight loss. Further, the modest reported changes in total medical and health services costs that were modeled can further inform decision making by local communities, employers, and insurers regarding the economic value and feasibility of similar lifestyle maintenance programs.

## Supporting information

S1 Checklist CHEERS checklist final. (DOCX)

S1 File Supporting data for manuscript. (XLSX)
